# Cranial Suture Mesenchymal Stem Cells: Insights and Advances

**DOI:** 10.3390/biom11081129

**Published:** 2021-07-31

**Authors:** Bo Li, Yigan Wang, Yi Fan, Takehito Ouchi, Zhihe Zhao, Longjiang Li

**Affiliations:** 1 State Key Laboratory of Oral Diseases, National Clinical Research Center for Oral Diseases, Department of Orthodontics, West China Hospital of Stomatology, Sichuan University, Chengdu 610041, China; libo.scu@foxmail.com (B.L.); wongyigan.scu@foxmail.com (Y.W.); 2 State Key Laboratory of Oral Diseases, National Clinical Research Center for Oral Diseases, Department of Cariology and Endodontics, West China Hospital of Stomatology, Sichuan University, Chengdu 610041, China; yifan@scu.edu.cn; 3 Department of Physiology, Tokyo Dental College, Tokyo 1010061, Japan; takehitoo@tdc.ac.jp; 4 State Key Laboratory of Oral Diseases, National Clinical Research Center for Oral Diseases, Department of Head and Neck Oncology, West China Hospital of Stomatology, Sichuan University, Chengdu 610041, China

**Keywords:** mesenchymal stem cells, cranial sutures, injury repair, Gli1, Axin2, Prrx1, Ctsk

## Abstract

The cranial bones constitute the protective structures of the skull, which surround and protect the brain. Due to the limited repair capacity, the reconstruction and regeneration of skull defects are considered as an unmet clinical need and challenge. Previously, it has been proposed that the periosteum and dura mater provide reparative progenitors for cranial bones homeostasis and injury repair. In addition, it has also been speculated that the cranial mesenchymal stem cells reside in the perivascular niche of the diploe, namely, the soft spongy cancellous bone between the interior and exterior layers of cortical bone of the skull, which resembles the skeletal stem cells’ distribution pattern of the long bone within the bone marrow. Not until recent years have several studies unraveled and validated that the major mesenchymal stem cell population of the cranial region is primarily located within the suture mesenchyme of the skull, and hence, they are termed suture mesenchymal stem cells (SuSCs). Here, we summarized the characteristics of SuSCs, this newly discovered stem cell population of cranial bones, including the temporospatial distribution pattern, self-renewal, and multipotent properties, contribution to injury repair, as well as the signaling pathways and molecular mechanisms associated with the regulation of SuSCs.

## 1. Introduction

Suture mesenchymal stem cells (SuSCs), a heterogeneous stem cell population, belong to mesenchymal stem cells (MSCs) or skeletal stem cells (SSCs), with the ability to self-renew and undergo multi-lineage differentiation. So far, research on MSCs or SSCs has been majorly conducted and focused on the long bone. In the field of cranial bone research, relevant studies are quite limited. Therefore, it is not until recent years that the stem cell population of the cranial region has ultimately been identified and isolated with several markers [1,2,3,4]. Studies have revealed that, unlike the well-established perivascular niche of SSCs in the long bone [5,6,7], stem cells of the cranial bone are generally located and confined within the cranial suture mesenchyme, subsequently defined as SuSCs [2]. To date, there are only four markers, to the best of our knowledge, that have been verified to be labelled SuSCs, including Gli1 [1], Axin2 [2], Prrx1 [3], and Ctsk [4]. Moreover, these four SuSCs subsets certainly share some common properties, whereas they are still mutually distinguished.

In the long bone, SSCs play an essential role in plenty of physiological processes, such as growth and development, life-long homeostasis, and fracture healing [8]. Similarly, as the major stem cell population of cranial bones, the physiological significance of SuSCs is undoubted and self-evident. As early as 2000, Opperman [9] proposed that sutures functioned as intramembranous bone growth sites and acted as the major sites of cranial bone expansion during postnatal craniofacial growth. Indeed, the suture serving as the growth site for cranial morphogenesis is the equivalent of the epiphyseal plate in the long bone [10]. Under normal conditions, new bone is formed and deposited at the edges of the osteogenic fronts (OFs) on both sides of the suture, while the cells within the suture stay undifferentiated, which endows the skull to enlarge evenly in coordination with the brain growth. In recent years, studies have demonstrated that the healing rate of the calvarial bone defects is inversely proportional to the distance between the cranial suture and injury site [11]. Additionally, the capabilities of regeneration and restoration vary from site to site in the calvarium [12], and the cranial sutures possess significantly stronger regenerative ability than the non-suture region of the calvaria [11]. Along with the identification and isolation of SuSCs, the above-mentioned experimental results have been well validated and elucidated. Nevertheless, our current knowledge and understanding of cranial sutures and SuSCs is still minimal and elusive. It remains to be further explored and clarified from the following perspectives, such as the participation of SuSCs in the growth and development of the skull, the exact function of SuSCs in the maintenance of homeostasis and local microenvironment, the precise role of SuSCs in calvaria injury repair, and the underlying regulatory mechanism. In particular, as for cranial bone repair and regeneration, it is of great significance to elucidate the cellular and molecular mechanisms and the source of reparative SuSCs and their progenies involved in the re-ossification and healing process. To this end, we aim to summarize the most up-to-date advances of SuSCs, the recently discovered craniofacial stem cell population, regarding the temporal and spatial distribution pattern, the cell biology characteristics, the essential role in cranial bone injury repair, and, most importantly, the signaling pathways and potential interplay mechanisms in the mediation and regulation of SuSCs.

## 2. The Anatomy of Cranial Sutures and Craniosynostosis

The cranial suture is a dense, fibrous tissue that connects the bones of the skull. With such unique immovable joints, also known as synarthrosis, the separated cranial bones are bound together as a rigid entity, supporting the craniofacial structures and providing the brain with a protective cavity. Only a tiny amount of movement is permitted at cranial sutures, which favors the elasticity and compliance of the skull.

### 2.1. The Anatomy of Cranial Sutures

**Figure 1:**
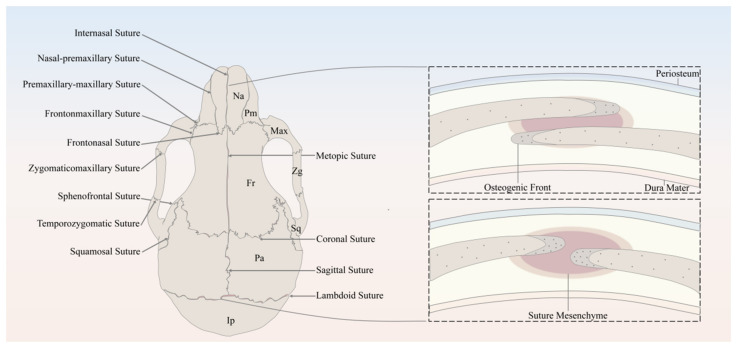
Schematic of the murine skull in the dorsal view, depicting the anatomy of cranial bones and sutures. Na, Nasal; Pm, Premaxilla; Max, Maxilla; Zg, Zygomatic; Sq, Squamosal; Fr, Frontal; Pa, Parietal; Ip, Interparietal.

The major sutures of the skull vault include the following: the metopic suture, also referred to as the frontal/interfrontal suture, extending from bregma to nasion, located between the two frontal bone plates; the sagittal suture extending from the bregma to lambda, located between the two parietal bone plates; the coronal suture extending from bregma to left/right pterion, located between the frontal bone plate and the left/right parietal bone plate; and the lambdoid suture extending from lambda to left/right asterion, located between the occipital bone plate and the left/right parietal bone plate (Figure 1).

It has been well established that the derivation of the skull vault comes from dual tissue lineages, namely, paraxial mesoderm and cranial neural crest [13,14,15]. As for the cranial sutures, not only do the sutures separate bones of different embryological origin, but they are themselves derived from different origins [15]. For instance, sutures derived from cranial neural crest include metopic sutures and sagittal sutures, while coronal sutures derived from paraxial mesoderm and the developmental origin of the lambdoid sutures remains unknown [13,15]. Differing in embryonic origin may lead to distinct capabilities of SuSCs in different sutures.

In spite of the discrepancy in anatomical locations and embryonic origins, cranial sutures have similarly fundamental features, which can be considered as a complex composed of four principal components [13], including the OFs of the approximating bone plates, the suture mesenchyme spanning the OFs, the overlying periosteum, and the underlying dura mater, which is also the outermost layer of meninges, namely, membranous coverings of the brain and spinal cord. To be noted, the OFs of the bone plates of the coronal and lambdoid sutures partially overlap each other, whereas the counterparts of the metopic and sagittal sutures abut end to end [13] (Figure 1).

Throughout the growth and development of the skull, the cranial suture mesenchyme remains unossified, which interposes between the OFs of the adjoining bone plates. The MSCs and osteoprogenitors residing along the OFs keep proliferating, subsequently differentiate into osteoblasts, and contribute to the new bone formation through intramembranous ossification [9,16], which happens with a direct differentiation into osteoblasts from MSCs and/or osteogenic precursors without assuming a chondrogenic fate. During the above-mentioned process, the osteoblasts will secrete a kind of extracellular collagen matrix called osteoid, which will then become mineralized and deposit at the leading edges of bone plates. As for the bone plates, they remain separated to allow the growth and expansion of the skull in concert with the growing brain [13].

### 2.2. Craniosynostosis

**Figure 2:**
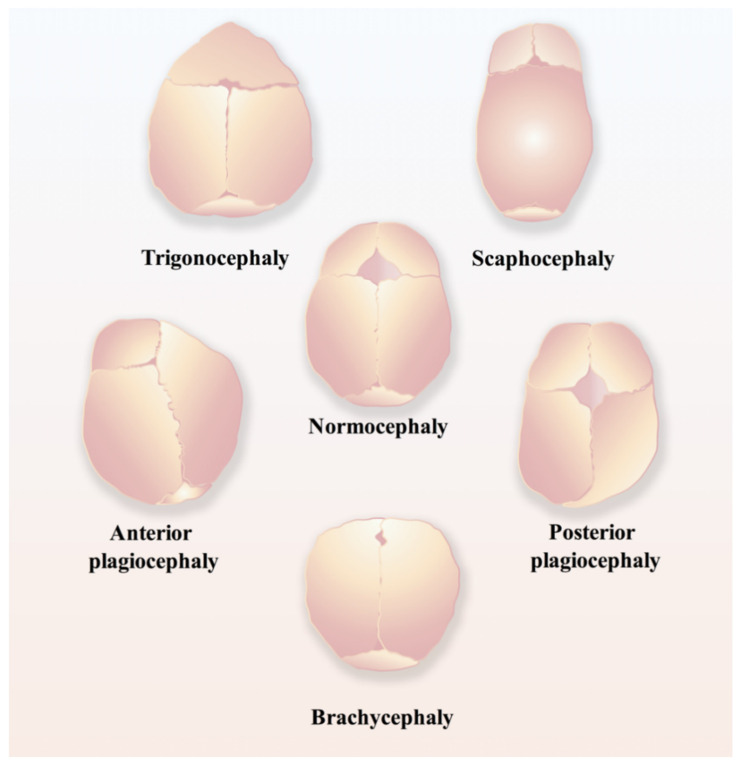
Schematic of different types of craniosynostosis, including trigonocephaly, scaphocephaly, anterior plagiocephaly, posterior plagiocephaly, and brachycephaly.

Regarding the time points of normal closure of cranial sutures, in humans, the frontal suture usually undergoes fusion within three to nine months after birth, while other cranial sutures will stay patent until adulthood [17]; in mice, the posterior part of the frontal suture will be fused by endochondral ossification within one month postnatally, while the anterior part of the frontal suture and other cranial sutures never fuse and remain patent throughout life [18,19]. Craniosynostosis (Figure 2), the premature fusion of the cranial suture, will hamper the normal development of the brain and often leads to the impairment of cognitive functions, sometimes even intellectual disabilities, due to increased intracranial pressure [16,20,21,22]. Currently, the only applicable therapeutic option for craniosynostosis is complex surgery, for instance, spring-mediated cranioplasty and minimally invasive strip craniectomy partial craniectomy followed by cranial molding orthosis (helmet) therapy, to correct the deformity and prevent its devastating sequelae [23,24], which are most likely to occur if appropriate surgical intervention is not conducted in time [24].

Numerous published studies have shown that craniosynostosis is associated with *TWIST1* mutation and *FGFR* mutation [18,20,25], and also relates to the premature loss of SuSCs [26], which perturbs the production of sufficient undifferentiated mesenchymal cells to maintain the suture patent [1]. More importantly, the aforementioned four distinctive markers of SuSCs also have a strong relationship with craniosynostosis. It has been found that *Twist1*^+/−^ mice with premature cranial suture obliteration phenotype have a severe reduction in the number of Gli1+ SuSCs regardless of whether the sutures are fused or remain patent. In addition, ablation of Gli1+ SuSCs by using *Gli1-Cre^ERT2^;DTA^flox/flox^* mice results in the typical phenotype of craniosynostosis, which is mediated via diphtheria toxin fragment A (DTA) under the inducible Cre-loxP system [1]. Other groups have found that loss-of-function mutations of *AXIN2* will cause excessive ossification in cranial sutures, leading to craniosynostosis in mice [27,28] and humans [29]. In contrast, DTA-mediated lineage ablation of Prrx1+ SuSCs in mice at postnatal day 28 (P28) or P7 did not cause any significant craniofacial phenotype, and only changes in the length of the femur and tibia were observed [3]. Recently, the transcriptional profile of the metopic sutures of *Twist1*^+/−^ and *Fgfr2^+/S252W^* mice have been unraveled with the advancement of single-cell RNA sequencing (scRNA-seq) technique, which enables one to decipher the spatiotemporal dynamics of suturogenesis at the single-cell transcriptomic level. It was uncovered that the major transcriptional changes of the above-mentioned two mouse models of suture dysgenesis were associated with angiogenesis and ribogenesis, respectively, whereas the cell subpopulations were not significantly altered [18]. Therefore, the high-resolution and comprehensive dataset of suture development indicates that transcriptional changes in the mouse model of craniosynostosis are model-specific. Besides, the discordance between mouse models of craniosynostosis and human patients should be taken into consideration as well, since the presence of cartilage and endochondral ossification is deduced to be involved in suture closure [14,30,31], which, in fact, has been observed in several human calvarial sutures under physiological conditions, especially with a high incidence in normal lambdoid sutures [32].

Revisiting history, researchers previously utilized rabbits to generate models of craniosynostosis, either occurring naturally [33,34,35] or inducing through immobilization of the suture with mechanical restraint (mostly via methyl-cyanoacrylate adhesive) [36,37,38], and thereafter, to investigate the treatment methods accordingly. Recently, a novel idea for the treatment of craniosynostosis has been brought up based on a rigorous murine study [21]. The researchers generated a rectangular defect over each of the fused coronal sutures in *Twist1*^+/−^ mice, then combined Gli1+ SuSCs from healthy donor mice with several different biomaterials collectively termed M-GM (GelMA:Matrigel:COL-I mixed at a ratio of 10:2:1) and implanted them together into the defect of post-craniectomy *Twist1*^+/−^ mice. Intriguingly, it turned out to regenerate the normal suture successfully since the newly formed coronal suture maintains its function and structure for nearly 1 year after surgery. This SuSC-based therapy not only corrects skull deformity and restores intracranial pressure to normal, but also reverses neurocognitive deficits caused by craniosynostosis [21], which possibly offers a paradigm shift in treating this devastating disease and is promising to be translated into human clinical applications.

## 3. Temporal and Spatial Distribution Features of SuSCs Subpopulations

**Figure 3:**
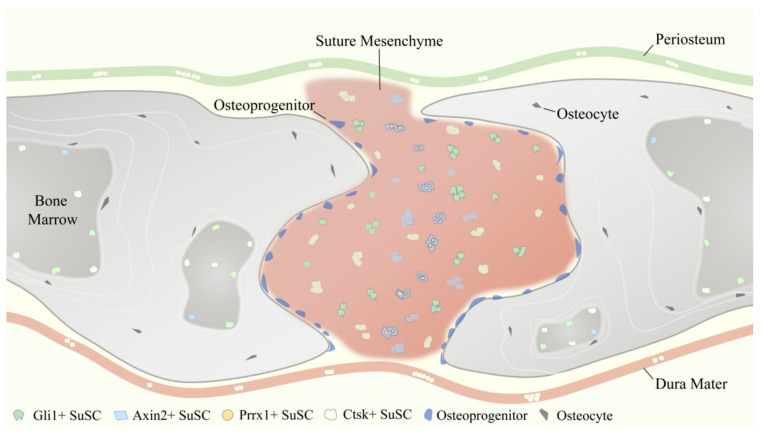
Temporospatial distribution pattern of four representative suture mesenchymal stem cells (SuSCs) subpopulations, including Gli1+ SuSCs, Axin2+ SuSCs, Prrx1+ SuSCs, and Ctsk+ SuSCs.

As aforementioned, reporter constructs expressed by Gli1 [1], Axin2 [2], Prrx1 [3], and Ctsk [4] have been identified to label SuSCs specifically. These four SuSCs subpopulations are physically tightly related but do not fully overlap (Figure 3), reflecting the heterogeneity of MSCs to some degree. Therefore, the use of multiple markers together might delineate and define SuSCs better, on which to date no study has been done.

### 3.1. Temporal and Spatial Distribution Features of Gli1+ SuSCs

Gli1 is an essential transcription factor of the Hedgehog (Hh) signaling pathway. Currently, it has been widely validated as a common and reliable MSCs marker in a variety of tissues and organs throughout the whole body [39], including but not limited to the skull [1], long bones [40,41], incisors [42,43], periodontal ligament [44], intestine [45], and respiratory tract [46].

From P0 to 1 month after birth (P30) in mice, which is equivalent to the early stage of growth and development in humans, Gli1+ SuSCs gradually restrict the cranial suture from a widespread distribution pattern and are ultimately confined to the suture mesenchyme [1]. Specifically, Gli1+ SuSCs can be detected in the periosteum, dura mater, and suture mesenchyme from P0 to P14, whereas from P14 to P30, Gli1+ SuSCs are almost merely observed within the cranial suture region. In addition, Gli1+ SuSCs are also observable lining on the inner surface of the cranial bone marrow cavity, even though the number is neglectable. After 8 months of lineage tracing, Gli1+ SuSCs and their progenies are distributed throughout the calvarium, detectable in cranial suture mesenchyme, periosteum, and dura mater. Moreover, quite a few osteocytes are also labeled, indicating that they derive from Gli1+ SuSCs [1]. Additionally, our research group carried out short-term (30 days) and long-term (1 year) lineage tracing and verified the suture-specific distribution pattern of Gli1+ SuSCs independently. Recently, a tissue clearing method, the PEG associated solvent system (PEGASOS) [47], has been applied to render craniofacial bones entirely transparent and to delineate the temporospatial distribution of Gli1+ SuSCs with deep imaging techniques [48]. It has been convincingly demonstrated that Gli1+ SuSCs are spatially associated with vasculature during the postnatal craniofacial development [48], which implies Gli1+ SuSCs may bear a resemblance to MSCs of long bones, for instance, residing in a peri-vascular milieu.

### 3.2. Temporal and Spatial Distribution Features of Axin2+ SuSCs

Axin2 is a direct transcriptional target of β-catenin, as well as a negative regulator of the Wnt/β-catenin (Wnt) signaling pathway [30]. Unlike Gli1+ SuSCs, Axin2+ SuSCs do not exhibit a diffused distribution pattern in the calvarium in the early stages of postnatal growth and development and are rarely detected in either the periosteum or the dura mater. Starting from P0, Axin2+ SuSCs are primarily located in the cranial suture and concentrated in the midline of the suture mesenchyme. After 1-month, 3-month, and 1-year lineage tracing, Axin2+ SuSCs and their progeny cells continue to accumulate without showing any sign of diminishing. Besides cranial sutures, Axin2+ SuSCs and their derivatives are also widely expanded and found within periosteum and dura mater over a long tracing period; some of the cells are embedded in the bone plate as osteocytes. Even more than 1 year of tracing, the Axin2+ SuSCs and their derivatives remain detectable and keep increasing in all calvarial sutures except the posterior frontal suture, which generally undergoes fusion in juveniles [2].

### 3.3. Temporal and Spatial Distribution Features of Prrx1+ SuSCs

Prrx1 is a transcription factor highly expressed during limb bud formation [49,50], and craniofacial development [50]. In terms of localization, Prrx1+ SuSCs have their own unique characteristics. To illustrate, Prrx1+ SuSCs reside exclusively within the posterior frontal suture, coronal suture, sagittal suture, and lambdoid suture, but are absent in other craniofacial sutures, periosteum, and dura mater. Interestingly, the number of Prrx1+ SuSCs will decrease with age. The quantitative analysis of 8-, 16-, 24-, and 32-week-old mice showed that the total cellularity or the cell density of the suture did not change with age; however, the population of Prrx1+ SuSCs displayed a significant reduction with age continuously. The total number of Prrx1+ SuSCs in the coronal suture decreased up to 75% from 8 weeks of age to 32 weeks of age [3]. This result may attribute to the fact that the Prrx1-expressing cells contain a large proportion of osteoprogenitors or transit-amplifying cells (TACs), while the percentage of the bona fide SuSCs is low. Last but not least, Prrx1+ SuSCs can express Axin2, which will increase upon the stimulation of the Wnt agonist. Thereby, the researchers postulate that Prrx1+ SuSCs are a subset of Axin2+ SuSCs [3].

### 3.4. Temporal and Spatial Distribution Features of Ctsk+ SuSCs

For decades, Cathepsin K (Ctsk) has been widely conceived as a classic marker of osteoclasts in the field of bone research [51]. However, in the past few years, it has been revealed that Ctsk can label the mesenchymal progenitors in the perichondrial groove of Ranvier [52]. More recently, another group identified Ctsk-expressing stem cells located in the periosteum of long bone and cranial suture via the scRNA-seq approach, together with lineage tracing and a series of rigorous in vitro and in vivo experiments to verify their ‘stemness’ [4]. Leaving long bones alone and focusing solely on the skull, at P15 and P32, Ctsk+ SuSCs and their progenies were not only presented in the cranial sutures, but also in the overlying periosteum, underlying dura mater, and bone marrow cavity of the calvarium, which was indicated by the visible signals of membrane-bound green fluorescent protein (mGFP). Further, fluorescence-activated cell sorting (FACS) analysis of P6 calvarial tissues found that the percentage of Ctsk+ SuSCs in suture (31.1%) was significantly higher than the percentage of Ctsk+ SuSCs in calvarial periosteum (4.37%), suggesting the enrichment of Ctsk+ SuSCs in the suture region [4]. Therefore, cells with an immunophenotype of Ctsk+ SuSCs existed predominantly in the suture mesenchyme, which was also consistent with the notion that cranial sutures contain mesenchymal progenitors that migrated to the periosteum as maturation [1,2]. Although the majority of this study focused on exploring and discussing Ctsk+ periosteal stem cells (PSCs) in the long bone, it still provides some valuable insights on Ctsk+ SuSCs and their properties.

## 4. Characteristics of SuSCs and Their Role in Injury Repair

In general, the subpopulations of SuSCs labeled and distinguished by Gli1, Axin2, Prrx1, and Ctsk have similar but not identical biological characteristics. They all possess a self-renewal ability and multi-lineage differentiation potential (excluding Prrx1+ SuSCs, which were only tested for osteogenic differentiation [3]), and participate in calvarial bone injury repair (excluding Ctsk+ SuSCs, which did not have direct experimental evidence showing their involvement in calvaria injury repair, albeit Ctsk+ PSCs is proved to contribute to long bone fracture healing [4]).

### 4.1. Biological Characteristics of Different SuSCs Subpopulations

According to the International Society for Cellular Therapy (ISCT), a standard set of stem cell criteria must be fulfilled for defining MSCs [53,54], including plastic-adherent ability when cultured in vitro, specific surface markers expression (such as CD73, CD90, and CD105), and trilineage (osteogenic, chondrogenic, and adipogenic) differentiation potential. Of note, this definition is based on cultured cells, and it remains largely unknown regarding the criteria applied to identify MSCs in vivo. Hence, several crucial biological characteristics of each subset of SuSCs are reviewed here accordingly, including self-renewal ability, multipotency, and the expression of various markers of MSCs or SSCs.

As for the capacity to self-renew, both Gli1+ SuSCs and Prrx1+ SuSCs are tested through long-term lineage tracing, which somehow reflects the self-renewal ability of labeled cells, but have not been discussed in detail [1,3]. Axin2+ SuSCs have gone through long-term lineage tracing without decreasing in number; meanwhile, EdU (5-ethynyl-2′-deoxyuridine) assay and Ki67 immunofluorescence pointed out that Axin2+ SuSCs were slow cycling in nature instead of active proliferating, indicated by their label-retaining property [2]. For Ctsk+ SuSCs, mesensphere assays were performed consecutively for three rounds, which aimed to critically evaluate the self-renewal ability in vitro. As a result, more than 60% of Ctsk+ SuSCs were able to form primary and secondary mesenspheres, whereas tertiary mesensphere formation was significantly reduced [4].

Concerning the potential of multi-lineage differentiation, Gli1+ SuSCs were capable of trilineage differentiation under induction in vitro [1]. When comparing the differentiation potential of Gli1+ SuSCs with bone marrow-derived MSCs (BMMSCs) from the same mice, researchers noticed that Gli1+ SuSCs were more inclined to differentiate into osteoblasts and chondrocytes because of their remarkably weaker adipogenic performance [1]. For Axin2+ SuSCs, kidney capsule transplantation studies demonstrated that Axin2+ SuSCs could form ectopic bone in vivo without any intervention and therefore possessed the osteogenic ability. In addition, the transplanted Axin2+ SuSCs were able to generate cartilage with the presence of bone morphogenetic protein 2 (BMP2), suggesting their chondrogenic potential [2]. Unlike Axin2+ SuSCs, Ctsk+ SuSCs could only undergo osteogenesis in vivo, which was revealed by the kidney capsule transplantation experiment, and could not give rise to cartilage, because Ctsk+ SuSCs were intramembranous-competent and predominantly orchestrated the process of intramembranous ossification. However, Ctsk+ SuSCs displayed clonal multipotency in vitro for differentiation into osteoblasts, chondrocytes, and adipocytes [4]. Prrx1+ SuSCs were only tested and verified for their osteogenic differentiation ability upon appropriate induction (recombinant mouse WNT3a) [3].

Regarding the expression of MSC or SSC markers, FACS analysis revealed that Gli1+ SuSCs and their derivatives highly expressed typical MSC markers, including CD44, CD73, CD90, Sca1, and CD146, but did not express CD34 [1]. Astonishingly, immunohistochemical staining showed that most Gli1+ SuSCs did not express CD44, CD73, CD90, Sca1, and CD146 in vivo. By microarray and real-time RT-PCR, it was found that Axin2+ SuSCs highly expressed Leptin receptor (LepR) and Gli1, while the expression levels of Nestin, Gremlin1, and CD146 were similar to Axin2− suture cell populations [2]. Similarly, it was detected by real-time RT-PCR that Prrx1+ SuSCs exhibited an elevation of Pdgfrα and CD146 compared with Prrx1− cells isolated from calvarial sutures [3]. As for Ctsk+ SuSCs, CD45^−^ Ter119^−^ CD31^−^ (Lin^−^) non-hematopoietic and non-vascular endothelial Ctsk-mGFP cells [55,56] were initially sorted by FACS, and subsequently, the expression of SSC markers was examined in the sorted Ctsk-mGFP cells. Finally, Thy1.2^−^, 6C3^−^, CD49f^low^, CD51^low^, CD200^+^, CD105^−^ Ctsk-mGFP cells were determined as Ctsk+ SuSCs, which did not express CD146 and Sca1 [4,57].

### 4.2. Contribution of SuSCs in Calvarial Bone Injury

SuSCs play an indispensable role in the injury repair and tissue regeneration of calvarial bone defects after birth [58]. Studies have shown that Gli1+ SuSCs were rapidly activated into proliferation within 24 h after an injury occurs. Two weeks after experimental injury, most of the infiltrated cells within the injury site were labeled, indicating their derivation from Gli1+ SuSCs. One month after experimental injury, the periosteum, dura mater, and osteocytes in the re-ossified region were labeled, suggesting that Gli1+ SuSCs contribute to calvarial bone defect repair [1]. Additionally, *Gli1-Cre^ERT2^;R26-ZsGreen^flox^* mice were induced and their calvaria (skull bone flaps containing the sagittal suture) were dissected under sterile conditions and transplanted into nude mice, which were used as the recipient mice with a calvarial window for placing transplants. It was found that the suture transplants integrated into the host bone and healing were achieved one-month post-surgery, with a significant number of cells within the periosteum, dura mater, and bone of the transplant strongly labeled. On the contrary, transplants not containing any suture tissue (with periosteum and dura mater preserved) from the same donor mice were transplanted and served as controls and ended up with poor healing and failure in generating new periosteum, dura mater, or osteocytes [1]. Thus, the cranial sutures and the resident Gli1+ SuSCs are the main sources of reparative cells functioning in calvarial bone injury repair; the periosteum and dura mater are either unable or insufficient to accomplish efficient calvarial bone regeneration. Similarly, Axin2+ SuSCs also respond to calvarial bone injury and promptly expand within the suture mesenchyme. Four weeks after experimental injury, a drastic expansion of Axin2+ SuSCs has been observed surrounding the skeletogenic suture mesenchyme. Further, Axin2+ SuSCs moved into the injury site and co-localized with Osx+ osteoprogenitors and Sost+ osteocyte, indicating their direct contribution to the injury repair of the skull. When Axin2+ SuSCs were isolated from *Axin2^Cre-Dox^;R26RlacZ* mice and directly implanted into the injury site, enhancements of the healing process were detected at two and four weeks after the operation. In comparison, neither transferring Axin2^−^ cells nor implanting without any cells serving as control did not show significant improvement [2]. As expected, 5 days, 10 days, and 30 days after experimental injury, Prrx1+ SuSCs and their progenies were found to contribute to the repair and regeneration of neural crest-derived (frontal) and mesoderm-derived (parietal) calvarial bones [3]. Plus, the parietal bone defects were unable to heal if the surrounding coronal and sagittal sutures were surgically removed concomitantly to the creation of the defect, while removal of the sutures away from the parietal bone defects did not affect the healing process [3]. Regarding Ctsk+ SuSCs, it has not been tested through the calvarial bone defect model to evaluate their performance in injury repair. However, based on the pivotal role of Ctsk+ PSCs in the process of long bone fracture healing [4], we speculate that Ctsk+ SuSCs should facilitate calvarial bone healing as well.

## 5. Signaling Pathways in the Regulation of SuSCs

**Figure 4:**
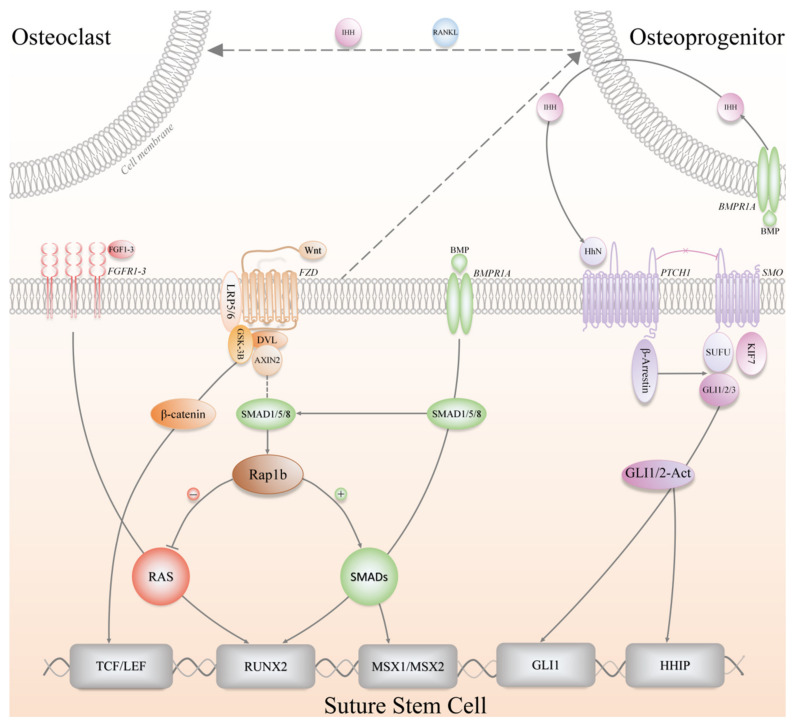
The signaling pathways involved in the regulation of SuSCs, including Wnt (Wnt/β-catenin), FGF (Fibroblast growth factor), BMP (Bone morphogenetic protein), Hh (Hedgehog) signaling pathways. IHH, Indian hedgehog; RANKL, Receptor activator of nuclear factor-κB ligand; BMPR1A, Bone morphogenetic protein receptor type 1A; FGFR, Fibroblast growth factor receptor; FZD, Frizzled; LRP5/6, Low-density lipoprotein-related receptors 5/6; DVL, Dishevelled; GSK-3B, Glycogen synthase kinase 3-beta; PTCH1, Patched 1; SMO, Smoothened; SUFU, Suppressor of fused; KIF7, Kinesin family member 7; TCF/LEF, T-cell factor/Lymphoid-enhancer factor; RUNX2, Runt-related transcription factor 2; MSX1/2, Msh homeobox 1/2; HHIP, Hedgehog interacting protein.

SSCs participate in skeletal growth and development, life-long homeostatic maintenance, and injury repair, providing the bones with a supply of osteochondroprogenitors cells [8]. Over decades, the above-mentioned physiological processes of long bone-derived SSCs or MSCs have been extensively studied, which are meticulously orchestrated by a variety of convoluted signaling pathways, such as transforming growth factor-β (TGFβ)/bone morphogenetic protein (BMP) signaling [59,60], parathyroid hormone (PTH) signaling [61], Wnt signaling [62,63], Hh signaling [42,64] and fibroblast growth factor (FGF) signaling [65,66], etc. These regulatory signaling pathways have a great deal of crosstalk in maintaining a stem cell niche, and therefore, they appear to integrate and function as a delicate network (Figure 4). Hitherto, the signaling pathways and molecular mechanisms associated with the regulation of SuSCs have not been well clarified yet, and relevant studies are limited with preliminary conclusions presented. Besides, plenty of the mechanistic findings in SuSCs were based on previously established concepts in SSCs of long bones.

In recent years, it has been proposed that Hh signaling regulates Gli1+ SuSCs, and researchers have emphasized that Indian hedgehog (IHH) from the OFs rather than Sonic hedgehog (SHH) plays a pivotal role in inducing the osteogenic lineage commitment of Gli1+ SuSCs, which has been validated by utilizing multiple genetically engineered mice, including *Ihh-LacZ* reporter mice, *Shh-Cre^ERT2^;R26-tdTomato^flox^* mice, and *Gli1-Cre^ERT2^;Smoothened^flox/flox^* mice (*Smo* ICKO) [1]. After tamoxifen induction, *Shh-Cre^ERT2^;R26-tdTomato^flox^* mice did not show any positive signal in the suture region, while *Ihh-LacZ* reporter mice demonstrated IHH+ cells locating in the OFs, flanking the suture and positive for Sp7 (Osx) and Runx2 [1]. When the Hh signaling pathway was genetically blocked, *Smo* ICKO mice did not exhibit any notable phenotype regarding the patency of the cranial sutures as well as the proliferation and differentiation of Gli1+ SuSCs [1]. However, it was found that all the craniofacial bones in *Smo* ICKO mice displayed reduced bone volume and severe osteoporosis after eight-month observation. Meanwhile, in vitro experiments have revealed that IHH treatment significantly upregulated Gli1 activity and enhanced osteogenic differentiation, whereas Hh inhibitor GDC0449 treatment significantly downregulated Gli1 activity and dampened osteogenic differentiation [1]. Thereafter, the same research group went one step further and discovered the interplay between Gli1+ SuSCs and osteoclasts mediated by BMP signaling and IHH, which helps maintain calvarial bone homeostasis and injury repair [67]. Specifically, BMP signaling stimulated Bmpr1a+ osteoprogenitor cells to secrete IHH, which in turn promoted the osteogenic differentiation of Gli1+ SuSCs; concurrently, BMP-mediated IHH signaling functioned synergistically with receptor activator of nuclear factor kappa-B ligand (RANKL) to stimulate osteoclast differentiation and resorption activity, thereby maintaining the morphology and function of cranial sutures [67]. Nonetheless, other investigators have found and reported contradicting evidence of SHH expression in the suture mesenchyme [68,69], especially in the midline region in a patched pattern. Moreover, the function of SHH is elusive in the context of cranial bones, though researchers postulated that SHH might be essential in maintaining suture patency and regulate intramembranous bone formation and cranial suture morphogenesis [69,70]. Furthermore, SHH might increase mesenchymal proliferation via the promotion of Msx2, and similarities are present between the expression of SHH, Msx2, and BMP during neonatal craniofacial suture development [70,71].

Given the fundamental roles of Wnt signaling in cell fate determination, it is not surprising that Axin2+ SuSCs and Prrx1+ SuSCs have been found to be governed by Wnt signaling [2,3]. The research group, which identified and isolated Axin2+ SuSCs for the first time, has demonstrated in a preliminary study that disruption of the genes encoding AXIN2 and FGFR1 (Fibroblast growth factor receptor 1) in mice would induce chondrogenesis and endochondral ossification within the cranial suture mesenchyme, resulting in abnormal cranial suture fusion and skull deformities [30]. Mechanistically, the in-depth analysis revealed that Wnt signaling directly controlled the skeletal progenitors by modulating their renewal and proliferation, and indirectly affected lineage specification by influencing the balance of FGF and BMP signaling pathways [30]. After the identification of Axin2+ SuSCs, this group continued exploring the Wnt, FGF, and BMP signaling network in regulating the osteogenic and chondrogenic differentiation of SuSCs, and discovered an essential effector, Rap1b, acting downstream of Axin2 as a signaling interrogator for FGF and BMP through proteomic approaches [72]. Taken together, the balance between FGF and BMP signaling is critical for the development of craniofacial skeletons and the determination of SuSCs fate, which is controlled by the Axin2-Rap1b-mediated Wnt signaling pathway [30,72]. Likewise, Prrx1+ SuSCs were also found to respond to Wnt signaling both in vitro and in vivo [3]. In brief, Prrx1+ SuSCs highly expressed *Dkk1* and *Sost*, two genes encoding inhibitors of the Wnt signaling pathway, under physiological conditions [73], which suggested that the inactivated Wnt signaling helped maintain the undifferentiated quiescent status of Prrx1+ SuSCs; by contrast, activation of Wnt signaling by recombinant mouse WNT3a treatment led to an increase of osteodifferentiation and overexpression of *Axin2* in Prrx1+ SuSCs [3].

The BMP signaling pathway has been demonstrated to be an essential regulator in cranial biology [59,74]. Recent evidence faithfully proved that BMP receptor type 1A (BMPR1A) maintains SuSCs properties in craniofacial development as well as craniosynostosis. Axin2+ SuSC-specific disruption of *Bmpr1a* in mice, namely, *Axin2^Cre-Dox^;Bmpr1a^flox/flox^* mice, resulted in precocious differentiation and aberrant ossification, leading to craniosynostosis, which initiated at the midline of the suture [10]. It has also shown that BMPR1A is a surface marker of both mouse and human SuSCs because BMPR1A+ SuSCs are capable of generating ectopic bone tissue [10]. Hence, more BMP-based lineage tracing studies can be conducted to evaluate the potential contribution of BMP to SuSCs at embryonic and postnatal stages. Incidentally, to the best of our knowledge, there is no literature reporting regulatory mechanisms of Ctsk+ SuSCs so far.

Altogether, due to the insufficiency of relevant studies and the entanglement of various signaling pathways, our current knowledge of the underlying modulatory mechanisms in SuSCs maintenance and regulation is still in its infancy. More high-quality research is expected in the future to explore and elucidate the cellular characteristics and molecular mechanisms of SuSCs.

## 6. Summary

In this review, we have summarized advancements in SuSCs, the newly identified craniofacial stem cell population, and provided valuable insights from the following prospects: temporal and spatial distribution pattern, the biological features of different subsets, the essential role in cranial bone injury repair, and the regulatory signaling pathways as well as their potential interplay. Overall, SuSCs are qualified for the modern, stringent definition and thereby are considered as bona fide SSCs. Detailed investigations focusing on SuSCs are of profound significance, especially at the level of constructing the conceptual framework of cranial biology and intramembranous ossification. However, due to the limited accessibility of SuSCs, it is unavoidable to encounter some clinical translation hurdles that restrict advances in skeletal regenerative medicine. As further investigation of SuSCs and stem cell niches continues, elucidation of the cellular and molecular mechanism underlying the regulation of SuSCs, SuSC-mediated regeneration, as well as the causal link between congenital craniofacial anomalies and SuSCs dysregulation becomes an urgent demand for harnessing the therapeutic power of this promising craniofacial SSC population. Hopefully, SuSC-based therapy could serve as a reliable biological solution in the treatment of skull defects and deformities in the near future.
